# Factors Affecting on Couple’s Decisions to Use Surrogacy: A Qualitative Study 

**Published:** 2019-12

**Authors:** Farzaneh Golboni, Amir Jalali, Mohammadreza Dinmohammadi, Ziba Taghizadeh, Paricher Nouri, Mohammad Reza Salahsoor

**Affiliations:** 1 Department of Midwifery, School of Nursing and Midwifery, Kermanshah University of Medical Sciences, Kermanshah, Iran; 2 Department of Psychiatric Nursing, School of Nursing and Midwifery, Kermanshah University of Medical Sciences, Kermanshah, Iran; 3 Department of Nursing, School of Nursing and Midwifery, Zanjan University of Medical Sciences, Zanjan, Iran; 4 Department of Midwifery and Reproductive Health, School of Nursing and Midwifery, Tehran University of Medical Sciences, Tehran, Iran; 5 Department of Anatomy, School of Medicine, Kermanshah University of Medical Sciences, Kermanshah, Iran

**Keywords:** Qualitative Research, Infertility, Decision-Making, Iranian Couples

## Abstract

**Objective:** Our study aimed to clarify of factors affecting decisions to use a surrogate mother can create broad knowledge of this concept.

**Materials and methods:** For This qualitative research, participants were selected through snowball sampling methods out of couples with a history of using surrogacy as an alternative treatment for having a child. As well, sampling continued until data saturation was reached. Finally, 23 persons participated in study (9 couples, 5 related persons). After selecting the participants and obtaining informed consent, in-depth and semi-structured interviews were conducted and most of them were recorded with participants’ consent. Then, all the interviews were analyzed using a conventional method.

**Results:** Content analysis of the statements condenced to 311 codes, 13 subcategories and 5 categories including the absence of parental role, perceived norm, hope for parenting role, mental challenge, and decision to use surrogacy were extracted.

**Conclusion:** The results indicated that numerous variables had an effect on decision-making process to use a surrogate mother, but the variable of hope for parenting role was an influential concept that not only interacted with other concepts but also caused optimism and motivation in families to decide in this respect.

## Introduction

One of the most important issues couples face in life is fertility. Therefore, it can be argued that childbearing can be among the most significant goals of family formation. Infertility disorders also exist in all countries, and about one fifth of couples across the world are infertile (1). Infertility has also influenced about 10-15% of couples in the US and 20% of the total population in Western countries (2). Considering Iran, one fourth of couples are encountered by primary infertility (3). Moreover, the causes of infertility are divided into two categories: feminine and masculine (4). 

Advances in medical sciences can also raise scores of new human, ethical, legal, and social issues whose emergence can sometimes restrict or hinder such progresses. In this respect, surrogacy is considered as one of the scientific developments in recent decades for infertility treatment among couples which has become a controversial issue in medical sciences given its unique aspects. In addition, this method as an assisted reproductive technique can have its own multifaceted consequences within religion and society (5). 

Surrogacy contract or surrogate mother considered as one of the infertility treatment methods as well as an assisted human reproductive technique is an agreement by which a woman accepts to carry a pregnancy and deliver a baby to applicant parents after childbirth (6). One of the important factors affecting this process as well as the spread of this method is attitude, viewpoint, and thinking manner of people involved in this phenomenon (7). Currently, there are not lots of studies carried out on the effects of surrogacy in society (8). By reviewing the related literature, one can find that concepts related to the meaning of family, needs in family, and impacts of availability of treatments have been investigated in few studies (9). With regard to the low level of information within society and few studies carried out in this regard, the effects and benefits of using surrogacy have similarly caused many problems for those who intend to use this method (5) because lots of clients cannot opt for treatment methods by themselves (7). Thus, better implementation of the decision requires knowledge of attitudes by the stakeholders as well as important factors affecting decision-making (7, 10). Accordingly, the best method to understand the reasons behind this treatment method is to delineate the issue from the words of these individuals that can be really contributing (11). With attention to checking the decision-making process and the effective reasons, qualitative research was chosen as the best method for this study (12). Within a qualitative research, a researcher analyzes statements expressed by participants in a study and reports their experiences regarding a phenomenon examined (12, 13). Within this method, life experiences and giving a sense to them can be conducted through a mental and systematic approach and in a real environment (12). Thus, the present study was to investigate factors affecting decision to use a surrogate mother among a group of clients using a qualitative content analysis.

## Materials and methods

This study is parts of qualitative study that done by content analysis method. This study started on July 2016 until Aug 2017. The research context in this study was Institute and other private and public infertility centers in Tehran and Kermanshah and offices as well as infertility centers in other cities conducting surrogacy. 

The participants in this study included all the clients with a history of using surrogate mothers and selected via snowball sampling method taking maximum variation in sampling into account. In this study, a total of 25 interviews were conducted with 9 couples (18 wives and husbands), clients’ family members (two mothers-in-law), three therapists, one female physician, and two midwives who had long experience in the treatment process. 

The inclusion criteria were different age ranges, male and female genders (husband and wife), appropriate physical and mental status during interviews, history of using surrogacy for having a child, willingness to participate in study, and ability to speak and completion of informed consent form. Sampling was also continued until data saturation was reached. Moreover, the researcher used important and influential individuals in family, physicians, midwives, and therapist nurses who had experience working in infertility centers and related ones to select the participants because they could play effective roles in the process of decision-making. Finally, the participants were selected from couples who had at least a history of using a surrogate mother from different age groups as well as a history of suffering from different causes of infertility. 

The main data collection method used in this study was an in-depth and semi-structured interview using open-ended questions. The main interviews with participants were in individual, in-person, and face-to-face formats within different times (morning, evening, and night) in a convenient place determined by couples, their family members (home, workplace, and infertility centers), and therapists (workplace) (by the first author). The interviews were also recorded given the participants’ agreement by voice recorder (one of the couples avoided recording their conversations, so the interview was transcribed manually). Besides, note-taking was used during interviews in a way that features such as voice tone, pronunciation of words, laughs, cries, and pauses by the participants during the interviews were recorded. The duration of the interviews varied considering the participants’ abilities. The average interview time in each session lasted between 60 and 90 minutes. To facilitate data collection during the interviews, some guided questions were employed. The guided questions included:

     1. How did you choose to use surrogacy? 

     2. How did you come to the conclusion to choose this treatment method? 

     3. Who did contribute to your decision-making? 

     4. In your opinion, what problems do individuals choosing this method face? 

     5. Who and how can someone help with making decisions to use surrogacy in families involved? 

For data analysis in this study, conventional content analysis method (12, 14) was used without using any software (by the first and second authors). In this respect, data analysis was conducted simultaneously with data collection. First, the recorded interviews were listened carefully for several times; then, the interviews were transcribed manually. At this stage, the entire texts of the interviews were handwritten in a careful manner and verbatim. After that, the transcribed interviews were reread and coded thoroughly. Generally, the interviews were repeatedly read, the authors were immersed into the data, the data were organized, and finally the initial codes were extracted for data analysis and fulfillment of the desired objectives of the study. Next, the initial codes were categorized and subcategories and categories were established through continuous comparison of the data. Finally, the relationships were discovered via comparison of the categories and subcategories.

To ensure the accuracy and the reliability of the data; four criteria of trustworthiness (credibility, confirmability, dependability, and transferability) proposed by Guba and Lincoln were used (12), Using interviews and field notes, the researcher collected the data. Long-term presence in the clinical environment, long-term involvement with the data, and a history of doing qualitative research also helped the researcher to immerse into the data to obtain a rich understanding of participants’ experiences. In addition; while presenting contact numbers to the participants after conducting each interview, they were asked to attend a meeting to review the results of the interviews; then, the researcher provided the results of the interview analyses to the participants in individual meetings, and finally asked them to compare the results with their own experiences (member checking). During interviews with the participants and through reviewing the questions and answers, the findings of the answers to the questions were confirmed by expressing the replies and their repetitions. In case of any ambiguity, the issues were discussed and analyzed via raising complementary questions to complete the details and to work out the ambiguities. Maximum variation was also considered in the sampling. 

To ensure that the data reflected participants’ experiences, peer checking was employed. In this way, the results at each stage of the research were presented to the supervisors and faculty members and their views and opinions were considered. By conducting continuous theoretical sampling, the researcher similarly tried to increase the explanatory power of the theory created with a greater variation in the context of the study and provide a comprehensive description of the decision-making process. In addition to the experiences and a history of conducting multiple qualitative research studies, the researcher also helped with resolving the deficiencies and bestowing prime importance to the study. 

Obtaining a letter of permission from Ethical committee of Kermanshah University of Medical Sciences (KUMS.REC.1394.451), a written informed consent was provided and then completed and signed by the researcher and the participants after a short and clear description. The participants were also asked about the recordings of their statements and such conversations were written down if they were not willing to record them. As well, the participants were ensured that their data will remain confidential and they will not be handed to those involved in the study. The participants’ names were also known to the researcher and those introducing them. Besides, the participants were informed that they could withdraw from the study at each stage of the research.

## Results

In this study, a total of 25 interviews were performed with 9 couples, 2 family members, and 3 therapists who had long experience in the treatment process (two participants were interviewed twice). Demographic characteristics of couples participating in research brought in table 1. Mean of age in females and males' clients was 34.44 ± 4.95 and 38.33 ± 6.51 years. Mean infertility duration for females was 7.22 ± 2.77 years and marriage duration was 7.55 ± 3.05 years. 

**Table 1:** Demographic characteristics of couples participating in research

**Coupl** **e**	**No**	**Sex**	**Age** **(Y)**	**Married ** **History** **(Y)**	**Infertility ** **History ** **(Y)**	**Kind of ** **Infertility**	**Cause of Infertility**	**Job**	**Education Level**
1	P1	F	36	5	5	Primary	Pelvic tuberculosis	Tailor	Diploma
	P2	M	40	5	-	-	-	Tailor	High School
2	P3	F	39	6	6	Primary	Severe uterine adhesion	Employee	Bachelor
	P4	M	41	6	-	-	-	Engineer	Bachelor
3	P5	F	35	11	8	Secondary	Ashkerman syndrome	House Wife	Diploma
	P6	M	26	11	-	-	-	Shopkeeper	High school
4	P7	F	36	6	6	Primary	Prematurity of the uterus	House Wife	Bachelor
	P8	M	41	6	-	-	-	General Physician	Doctoral
5	P9	F	36	8	8	Primary	Endometriosis	House Wife	Diploma
	P10	M	44	8	-	-	-	Employee	Master of science
6	P11	F	34	12	12	Primary	Genital tuberculosis	House Wife	Diploma
	P12	M	45	12	-	-	-	Shopkeeper	Diploma
7	P13	F	22	4	4	Primary	Prematurity of the uterus	House Wife	Diploma
	P14	M	29	4	-	-	-	Employee	Bachelor
8	P15	F	34	5	5	Primary	Severe uterine adhesion	House Wife	Bachelor
	P16	M	38	5	-	-	-	Shopkeeper	Diploma
9	P17	F	38	11	11	Primary	Endometriosis	Employee	Bachelor
	P18	M	41	11	-	-	-	Employee	Bachelor

Y Year, P Participants

Analysis of the statements spelled out by the participants boiled down to 487 initial codes and finally 311 codes, 13 subcategories, and main categories including the absence of parental role, perceived norm, hope for parenting role, mental challenge, and decision to use surrogacy by continuous reviews and removal of duplicated codes (Table 2). 

***The absence of parental role:*** Couples always compare themselves with others and consider absence of a child and infertility as a deficiency and a gap in their parenting roles. Thus, frequent treatments and hopes that do not regularly come true are among the factors contributing to this parenting gap. Statements from the participants in this respect included: *Well, defects are with us who cannot have our own children. All individuals can do it but we cannot fulfill it because we suffer from some disabilities* (P.3). 

*I did not have any problems with infertility from the beginning, but I was annoyed by what others were saying…whenever you go they say that you are 40 years old but you do not have a child* (P.13). 

**Table 2:** Categories and subcategories of decision-making process for using surrogacy

**Categories**	**Subcategories**	
The absence of parental role	Having no child	

Perceived norm	Cultural context of family	Attitude
		Interactions
		Family traditions and customs
	Social conditions	Social challenge
		Social interactions
Hope for parenting role	Pleasure of association of having a child	
	Attempts for childbearing	
	Hope	
Mental challenge	Fear of doing surrogacy	
	Finding a reliable person	
	Beliefs	
Decision to use surrogacy	Mutual support among couples	
	Avoiding pressure from family	
	Legal actions on individual choices	

*I always think that I have not been a perfect woman, I could give birth to a baby but I always thanks God for everything I have* (P.1). 

***Perceived Norm:*** This main category was derived from the sum of the categories of cultural context of family and social conditions. 

***Cultural Context of Family:*** In the words of the clients, it was clearly seen that the cultural context of family was one of the most important factors influencing selection or non-selection of the given method. Family rebukes and interferences, disgracefulness of surrogacy, and even unawareness of family about surrogate mothers were cases that brought about many problems. In addition to family culture, use of surrogacy was contrary to religious rules according to religious standards and the cultural context of family especially in families with low knowledge and awareness of surrogacy was practically a very important deterrent. In this regard, some of the clients’ statements were as follows: *If my family especially my mother-in-law gets to know about it, she will make me miserable out and will say that the child is not yours, but that is somebody else’s* (P.7). 

*My problem is with my husband’s family and his relatives. They cannot accept this issue at all. These days they ask my husband to get divorced and say that his wife is unable to have a child* (P.11). *They told me to think about surrogacy… they could not see how terrible I felt* (P.1). 

***Social Conditions:*** Social conditions are among the variables that overshadow couples in terms of their choice of using a surrogate mother. In their interactions with social environment, as well as friends and acquaintances; couples are always impressed with their views and attitudes. Given that the use of surrogacy has not yet been fully described in Iran and most people are unaware of it, couples are also very concerned about coping with society because of their decisions and they are constantly challenged. In addition, religious and legal principles and rules governing society are among other important issues that pose challenges for couples. Inappropriate awareness among those in society, couples, and their families can also double the given worries about religious rules and legal issues associated with parenting which can deprive them of having children using this method. In this regard, some sayings of the clients were as follows: *He has not still given a certain answer about his acceptance … he says that it is really a shame if our neighbors find about that. I do not know how to hide this issue from others! My husband is terribly conjured and does not like others understand this issue and josh at him … he said that we need to do something to hide it! *(P.7). *My only problem is with society which makes the conditions really difficult. It is easy to realize this issue in Iran; however, I accepted this alternative method *(P.16). *The main problem is finding someone to do it, I mean a woman who can be trusted … this issue is really annoying *(P.2). 

***Hope for Parenting Role:*** This variable was associated with all the categories and it can be said in some ways that it was the main and central variable of decision-making process to use surrogacy. In all cases of failures and discomforts, the only variable making these couples hopeful towards future was hope for parenting role. This category was the result of three subcategories of pleasure of association of having a child, attempts for having a child, and hopefulness. In the statements and words of the participants, one could clearly see that faith and belief in God as well as hope and need for having a child were among the most important factors helping couples tolerate all financial and mental problems as well as familial and social sufferings and consequently have a sense of beauty towards the pleasure of association of having a child. In this regard, some statements of the participants included: *You almost always like to have a child. All the couples I see have children … I love children. My husband said that if we do it, we can raise our own child instead of an adopted one *(P.1). *I think that my husband likes to have his own child. Last year when the issue of a surrogate mother was raised, I suggested adopting a child… but he said that he did not like to adopt someone else’s child *(P.9). *I always think that if I had a child, he or she could be 3 or 4 years old. I could buy some items for my child or take him or her out with me ... but, God is really big. If God will, everything will be OK* (P.14). 

***Mental Challenges:*** Following the stability and acceptance of infertility, mental challenges always put couples in a state of tension and psychological-mental stress. Accordingly; financial, legal, and social issues of this activity, probable failures, awareness by relatives, and even understanding of children about how they were born were also among cases putting couples under pressure; however, hope for a parenting role and positivity i.e. belief in God and the point that the problems could be resolved over time could always make couples decisive to use surrogacy. Some statements from the participants were as follows: *Most couples are very confused and disturbed at first. Many of them get initially nervous and do not accept, but their needs for having a child makes it possible to come back and ask lots of questions and seek for help* (P.1). *I am afraid that my child might think that he or she is not ours or others might say nonsense words that I am not his or her mom… but I always do not care, I say that is all right, and the child can change everything* (P.12). *Three months ago, my husband and I reached an agreement to act for surrogacy. My husband used to say that he did not want a child but these days he tells that we can at least raise our own child* (P.9). 

***Decision to Use Surrogacy:*** Eventually as hope and positivity of having a child overwhelms fears and concerns, couples can believe that the only way has been to use a surrogacy contract. So, at first, they began to hide the issue from their relatives, and many of them change their place of residence for some time. Couples also come to the conclusion that they only have each other and they must support each other and go along with this difficult path, use legal advice to do this correctly, and try to observe important factors in choosing the donor and careful implementation of this process. Some sayings of the participants included: *My husband said that we had to do it together, and there was no need to talk about that with our parents, sisters, brothers, and no one else* (P.3). *My mother and my sister know about this issue but my husband did not talk about it to his family. He does not like to let others know about it. He said that I needed to be careful and not to speak a word because he would not forgive me forever *(P.7). *To be assured, we consulted with a lawyer; we also signed a contract and agreed upon taking the baby at the hospital* (P.14).

## Discussion

The purpose of this study was to explain the factors affecting decisions to use a surrogate mother through a qualitative method. Hope for parenting role was considered as one of the influential variables described by these individuals. Advances in infertility treatment techniques and widespread variation of these methods have also led to an increase in expectations for fertility in couples who have not had any children despite numerous surgical and pharmaceutical treatments (15). Besides, studies have shown that religiosity and religious beliefs can affect the way in which patients define and shape their behavioral patterns in the domain of health for the use of medical treatments (16). These components can similarly affect selection or non-selection of choosing fertility treatment methods. In an investigation conducted in 2002, it was found that more religious people were less likely to use assisted reproductive techniques than others to solve their infertility problems (17). The important and influential factors in the difference between results of this research and other studies are social culture, context and attitudes. Because beliefs and views of individuals are formed from customs and social interactions and religious beliefs that can vary in different communities.

The absence of parental role was another concept delineated in this regard. Accordingly, couples considered having no children and infertility as a deficiency, and took the repeated failures of treatment into account as factors that could make such a feeling in them stronger, which was by itself a reason for their request for help. Researchers have also reported the occurrence of impulsive behaviors and distressed pressures, depression, sense of helplessness, the absence of parental role, worthlessness, as well as anxiety and stress among infertile individuals (18). Other studies have also highlighted the tendency of men and women to be a parent as an important factor for using surrogacy (19). Furthermore, mood disorders are common in infertile men and women; thus, obtaining a successful result from treatments can reduce such problems (20). 

Cultural context of family was similarly one of the other concepts addressed by the study participants. The findings in this study revealed that cultural context of family could have significant effects on decision-making by couples. Family traditions and customs as well as their attitudes and interactions, and even family structure were regarded as important factors in this process. In this regard, various studies have highlighted the effects of family on important decisions made in life (8, 21). One of the important issues affecting mental health status of these individuals is attitudes by friends and those around them and generally attitudes in society towards a surrogate mother. Thus, lack of social support in the process of using a surrogacy contract due to public attitudes resulting from unawareness of this issue can pave the ways for vulnerability (22). 

Social conditions were also another variable affecting this process. Obviously, social pressures to have children can lead to mental, physical, and social sufferings especially for women whose maternal role forms up an element of their base and identity and having a child can be taken into account as a source of women’s power in family and society. It is natural that if these individuals lose such an identity; they can be affected with mental, personality, and social trauma (9, 23). Using surrogacy among women who cannot get pregnant due to medical problems can be a selective method (9). This method can help couples but it can be accompanied by numerous problems; for example, this method can have a lot of physical and mental stress for couples. A surrogacy contract can be also accompanied by social and psychological difficulties. In some societies, this issue can be considered as a stigma; moreover, couples have to keep their privacy and anonymity which can create a negative environment for them affecting their internal and external relationships and consequently cause isolation (24). Among the factors mentioned in this domain are legal and religious issues in societies regarding the use of this method that can lead to its selection or non-selection. For example, in a study conducted in 2009, experiences by some individuals about ethical issues were examined which pointed to the fact that only Sunni jurists had rejected using surrogacy and Shia ones had confirmed this method just for couples legally married and under certain conditions (25). In another study in 2003, different results were obtained. In this respect, the findings revealed that couples had shared the issue of using surrogacy with their families and friends without any worries and stress and they were also decided to talk about it with their children after they were grown up. Moreover, couples in this study considered this method as a positive experience and suggested it to other infertile ones (26). 

Mental challenges were also described as important and effective concepts by people. Using a surrogate mother can lead to various issues which can lead to mental challenges in couples whose purpose is to use this method. For example; if a surrogate mother is chosen from strangers or even from familiar individuals, it can lead to mental challenges in couples. It is also said that surrogacy using a known surrogate mother can significantly make family dynamics complicated. Thus, use of familiar surrogate mothers is prohibited. Furthermore; in a study, all couples had talked about this issue considering the use of this technique with their family and had not kept this issue as a secret for themselves (5, 23, 26). In another study, some mothers believed that they were not prepared to make others aware of this issue due to their cultural and environmental backgrounds (9). 

There were several limitations in this study. The small number of participants as well as lack of existing information and low cooperation by fertility and infertility centers led to difficult and long-term sampling. Much correspondence was also made by Kermanshah University of Medical Sciences with the given centers and finally a number of couples were selected by snowball sampling method. Low cooperation by some couples also created more problems. The study samples were similarly selected from the cities of Tehran and Kermanshah. The interviews were also conducted in locations determined by the participants. Thus, majority of interviews were performed in their place of residence and in some cases they were performed in parks. Some couples were unwilling to record their voices, so they were explained and ensured that the recordings will remain confidential which met their consent. Ultimately, two couples rejected to do so and their interviews were transcribed manually by the interviewer and a colleague.

## Conclusion

Finally, it was concluded that numerous variables had effects on the process of decision-making to use surrogacy but the concept of hope for parenting role was an effective variable which not only interacted with other concepts but also created hope and positivity in families to make such a decision.
